# Review: the risks of spray adjuvants to honey bees

**DOI:** 10.1093/jisesa/iead100

**Published:** 2023-12-06

**Authors:** Brandon Shannon, Hongyoung Jeon, Reed M Johnson

**Affiliations:** Department of Entomology, The Ohio State University, 1680 Madison Avenue, Wooster, OH, USA; Application Technology Research Unit, Agricultural Research Service, United States Department of Agriculture, 1680 Madison Avenue, Wooster, OH, USA; Department of Entomology, The Ohio State University, 1680 Madison Avenue, Wooster, OH, USA

**Keywords:** honey bee, adjuvant, risk assessment, surfactant, pesticide

## Abstract

Pesticide applications are often made as tank mixes containing multiple pesticide products and may include spray adjuvants to enhance pesticidal activities. The primary aim of adjuvant products is to increase the spreading and sticking of spray droplets and to increase the penetration of active ingredients through the cuticles of leaves or targeted pests, which can reduce the amount of active ingredient needed for effective pest control. Adjuvants are made up of compounds drawn from the “inert ingredient” list maintained by EPA but are identified as “principal functioning agents” when used in adjuvant products. These inert compounds do not undergo the same testing and risk assessment process that is required of pesticide active ingredients and generally have no mitigation measures that prevent application onto crops during bloom at times of day when bees are foraging. Honey bees (*Apis mellifera;Hymenoptera:Apidae)* are at an increased risk of exposure to adjuvant tank mixtures while providing agricultural pollination services. Colony losses attributed to pesticide applications thought to have low risk to honey bees have been reported, highlighting the need to better understand the toxicity of adjuvants included in pesticide tank mixtures. This review summarizes current literature on the risks posed to honey bees by agricultural adjuvants and tank mix combinations of adjuvants with pesticides. Based on the current state of knowledge, we make recommendations to pesticide applicators, product manufacturers, regulatory agencies, and researchers regarding adjuvant toxicity to honey bees with the goal of reducing risks that adjuvants pose to honey bees and other beneficial insects.

## “Inert Ingredients” in Pesticides and Adjuvants

An adjuvant is any compound intended to improve the handling or application characteristics of a pesticide application to enhance pesticidal activity (Young et al. 2016), which can potentially reduce the amount of active ingredient that is necessary for pest control. Adjuvants are included in pesticide formulations or can be separate products that are added to a pesticide tank mix (Tu and Randall 2003). The “principal functioning agents” that provide the desired function in an adjuvant product are drawn from the list of “inert ingredients” maintained by USEPA and consist of the same or similar compounds used as formulation components in traditional pesticide products (USEPA 2013a). While 51.6% of agricultural adjuvants were components in formulated pesticide products in 2021, standalone adjuvant products are projected to be the fastest growing segment of the agricultural adjuvant market (MarketsandMarkets 2022). Adjuvants may also be included in pesticides used within honey bee colonies to control common bee pests such as small hive beetle, *Aethina tumida* (*Coleoptera*: *Nitidulidae*) (Cuthbertson et al. 2013), and the *Varroa destructor* (*Mesostigmata*: *Varroidae*) mite (Rosenkranz et al. 2010), among others (Pasho et al. 2021). The global agricultural adjuvant market, which includes both pesticide formulation components and standalone adjuvant products, valued at 3.7 billion USD in 2022, is expected to increase by 5.2% annually to 4.7 billion USD by 2027 (MarketsandMarkets 2022), demonstrating the need to better understand the risks posed by inert ingredients to honey bees. The objective of this review is to summarize the literature on adjuvant toxicity to honey bees and provide guidance to pesticide applicators, manufacturers, regulatory agencies, and beekeepers for coordinated efforts to minimize risks to honey bees from pesticide applications.

In the United States, tests of pesticide active ingredients are required prior to product registration and sale. The pesticide label lists the exact chemical compound and composition of active ingredients included in the formulated product. There are federal laws for bee hazard labeling intended to mitigate the impacts of pesticide application to bees (USEPA 2013b). In California there are state laws that prevent pesticides harmful to bees from being applied in daytime hours during bloom (State of California – California Code of Regulations 2022). However, as adjuvants are considered inert ingredients, these compounds do not undergo the same testing and risk assessment that is required of active ingredients (Tu and Randall 2003, USEPA 2013c), though Quantitative Structure Activity Relationship (QSAR) modeling is generally performed for risk assessment prior to product registration (Manibusan et al. 2023). Adjuvants typically have no mitigation measures that prevent application onto crops during bloom at times of day when bees are foraging (Straw et al. 2022). The exact chemical composition and relative proportions of most adjuvant compounds included in pesticide formulations and standalone adjuvant products are considered a trade secret. Therefore, they are rarely listed on pesticide labels and only listed under broad categories on spray adjuvant labels (Fishel 2010, Weinhold 2010, Straw et al. 2022).

The same compounds that are inert ingredients in pesticide products often also serve as principal functioning agents in standalone adjuvant products. These can be classified by function, chemistry, or source (vegetable or petroleum oil derived), and different products contain different adjuvant compounds to serve different purposes. The function of these compounds can include pH buffering (Green and Hale 2005, Sobiech et al. 2020), anti-foaming (McMullan 2000, Green 2001, Witt 2001, Tu and Randall 2003), drift reduction (Holloway 1994, Zhu et al. 1997, Butler Ellis and Tuck 1999, Al Heidary et al. 2014), enhanced adhesion to leaves (Reeves 1993, Bergeron et al. 2000, Mulrooney and Elmore 2000, Green 2001, van Zyl et al. 2010), reduction of surface tension to increase the wetting, spreading, and penetration of a pesticide (Schönherr and Baur 1994, Penner 2000, Xu et al. 2010, Arand et al. 2018, Räsch et al. 2018, Gao et al. 2019, Li et al. 2019, 2021, Pan et al. 2022, Song et al. 2022), and other functions (Young et al. 2016). The 5 main chemical classes of principal functioning agents listed on spray adjuvant labels include (i) nonionic surfactants, (ii) crop oil concentrates, (iii) modified seed oils, (iv) organo-silicone surfactants, and (v) and hydrocolloid polymers (Abbott et al. 2021).

### Nonionic Surfactants

**Fig. 1.**
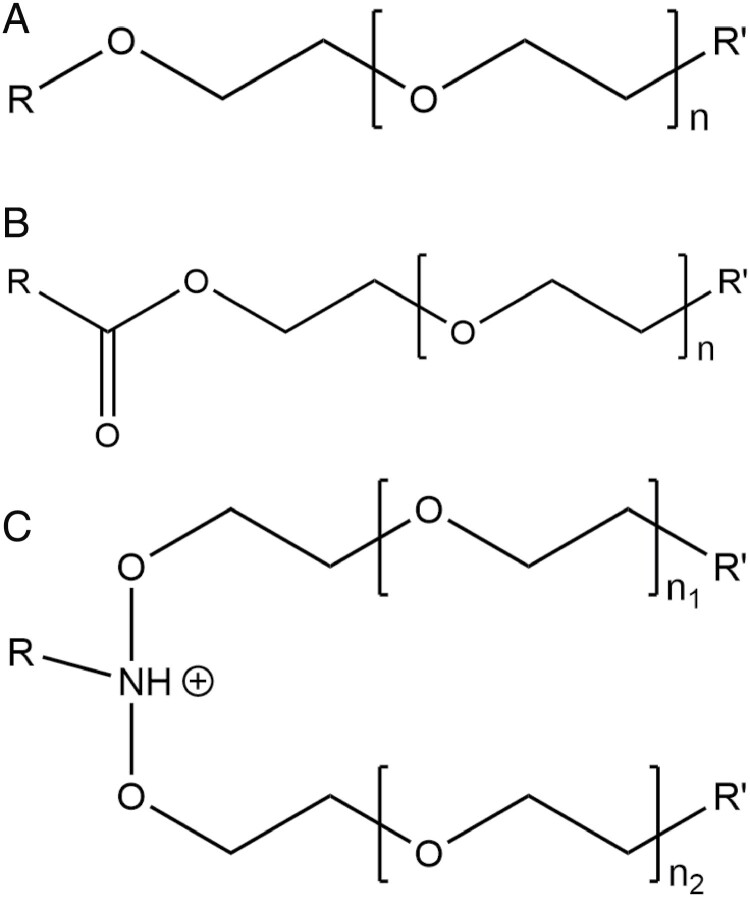
Generalized chemical structure of alcohol ethoxylates (A), fatty acid ethoxylates (B), and amine ethoxylates (C). For all groups, the hydrophobic *R* group head can be a variable carbon chain or ring structure and the length of the hydrophillic polyethoxylate tail, *n*, can be varied to provide the desired chemical or physical properties. The *Rʹ* and *R″* groups can be modified to change the cap of the hydrophillic polyethoxylate tail and is most often a hydroxyl group, but methyl and acetyl caps are also common. Adapted from (A) PubChem (2023a), (B) Stockburger (1979), and (C) Alexander and Richter (1999).

Nonionic surfactants are the most diverse chemical group and contain multiple subclasses. One sub-class of nonionic surfactant, the polyethoxylates, encompass primarily alcohol ethoxylates, fatty acid ethoxylates, and amine ethoxylates. These are made up of a hydrophobic head consisting of an alkyl or aryl alcohol, a fatty acid, or an amine bonded to a hydrophilic tail formed from ethylene oxide (Fig. 1). The chemical group of the head can be varied, the molar ratio of ethylene oxide added can be changed, and the ethoxylate cap can be modified to adjust the physical and chemical properties of the product. Most organic ethoxylates are classified as nonionic surfactants, however, some amine ethoxylates are classified as cationic surfactants due to the positively charged amine group, although cationic surfactants are not commonly used in agricultural applications (Rapp 2017). Other subclasseses of nonionic surfactant formulations include pinene or polyterpene compounds that are typically added to increase emulsification of oils.

### Crop Oil Concentrates and Modified Seed Oils

**Fig. 2.**
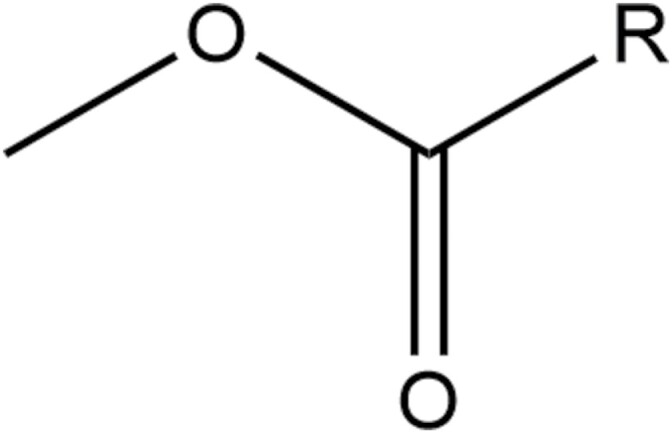
Generalized structure of a fatty acid methyl ester, where *R* is typically a saturated or unsaturated fatty acid carbon chain, with typical chain lengths between 14 and 24 carbons, more commonly as an even number (Hamilton 1993). Adapted from PubChem (2023b).

Crop oil concentrates and modified seed oils are mainly derivatives of naturally occurring plant oils, but also include petroleum-derived oils such as mineral oil. Crop oil concentrates include triglyceride oils and hydrocarbon oils, which are primarily isoalkanes, though petroleum-derived aromatics and naphthenes can be used to enhance product handling properties (Hamilton 1993). The most common type of modified seed oil is fatty acid methyl esters (Fig. 2), with a lipophilic tail and hydrophilic head. The chain length of the fatty acid can be modified to give the desired chemical and physical properties, but lengths between 14 and 24 carbons are typically used, with even numbers being more common than odd numbered chain lengths (Hamilton 1993). These are also often formulated with other classes of adjuvants, such as organo-silicone surfactants.

### Organo-Silicone Surfactants

**Fig. 3.**
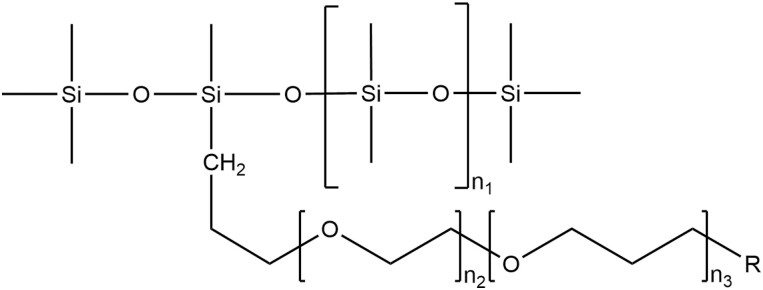
Generalized chemical structure of organo-silicone surfactants included in adjuvant and pesticide formulations. The hydrophobic siloxane head can be modified by changing the values of *n_1_* and *n_2_* and the hydrophillic ethoxylate tail can be modified by changing the values of *n_3_* and *n_4_* to give the compound the desired chemical and physical properties. Typically *R* is a methyl, acetyl, or hydroxyl group. Adapted from Stevens (1993) (Stevens 1993, Chen and Mullin 2013).

Organo-silicones are often considered “super-spreaders” and can decrease surface tension and increase spreading on waxy surfaces even when used at rates as low as 10% of traditional surfactant use rates. Organo-silicone surfactants can be ring-structured, but most are chain-structured with a dimethylsiloxane polymer and a terminal or side organic reactive group (Fig. 3). The dimethylsiloxane structure can be linear or branched and the organic reactive group can vary with corresponding changes in chemical and physical properties (Adams 1991, Stevens 1993, Chen and Mullin 2013). Most organo-silicones are nonionic surfactants, but the organic reactive group can be modified to become ionic (Adams 1991). Most commonly 3 dimethylsiloxane units form a trisiloxane backbone, but other siloxane polymers are also common.

### Hydrocolloid Polymers and Other Adjuvants

**Fig. 4.**
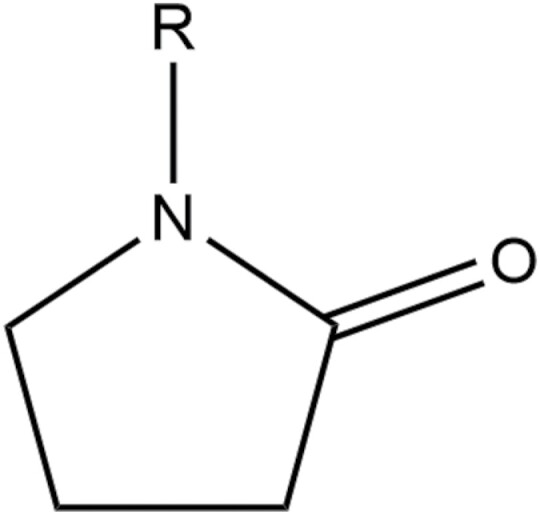
General chemical structure of N-alkyl-2-pyrrolidone, an emulsifier and antifreeze agent added into adjuvant and pesticide formulations. The *R* group can be modified to achieve the desired chemical and physical properties, but is most commonly a methyl, octyl, or dodecyl group (Mullin et al. 2015). Adapted from Mullin et al. (2015) and PubChem (2023c).

Hydrocolloid polymers are a diverse group of long-chain polymers that form viscous dispersions in water, which is intermediate between a true solution and a suspension and exhibit the properties of a colloid (Milani and Maleki 2012). They can be modified to add hydroxyl groups to increase hydrophilicity. Examples of these include polysaccharides such as agar, cellulose derivatives, gums, pectins, and starches or proteins such as gelatin (Milani and Maleki 2012). In addition to hydrocolloid polymers, other adjuvants are used as co-solvent antifreezes and emulsifiers such as butanol, diethoxol, methylcyclohexanone, alkylpyrrolidones, propylene glycol, and xylene (Mullin et al. 2015). One of the most common of these is N-methyl-2-pyrrolidone, in addition to N-octyl and N-dodecyl pyrrolidones (Fig. 4).

While there are many different compounds used as adjuvants, they all share the goal of enhancing the efficacy of the active ingredients included in a pesticide application. Adjuvants can decrease the total quantity of active ingredient required for effective pest control, with benefits for both human and environmental safety. However, this apparent benefit of adjuvant use could be self-sabotaging if these chemicals carry their own risks to pollinators and the environment.

## Honey Bee Exposure to Adjuvants through Pollination Services

To understand the risk adjuvants pose to honey bees, it is important to understand the likelihood, concentration, path, and duration of exposure associated with different routes as well as the inherent toxicity of adjuvants and potential interactive effects between the multiple components present in tank mixtures to honey bees. Beekeepers provide commercial pollination services to over 100 commercial crops in North America (Hristov et al. 2020), with an estimated contribution of 12 billion USD to the US economy (Calderone 2012, Khalifa et al. 2021) and 235–577 billion USD globally (Potts et al. 2016). Honey bee pollination is especially important during almond bloom in California, where approximately 500,000 ha of almond orchards required an estimated 2.6 million honey bee colonies for pollination services in 2021, representing 90% of the 2.92 million total managed honey bee colonies in the United States (Goodrich et al. 2021). Through their service in the commercial pollination industry, honey bees have the potential to receive higher exposure to agrochemicals than nonmanaged pollinators because they are transported to multiple blooming crops throughout the year that are managed with pesticides. For example, colony losses have been observed during and after almond bloom in California, where a survey found that 19% of beekeepers observed lethal effects and 56% observed sublethal effects in their colonies that they attributed to pesticide exposure (Goodrich et al. 2021). However, this may be an underrepresentation of the effects of pesticides and adjuvants, as the rapid effect of surfactants makes it more likely for bees to die in the field compared to traditional pesticide effects that would be observed at the entrance of hives by beekeepers (Goodwin and McBrydie 2000).

The ecological persistence of adjuvants is poorly understood, so the level of exposure beyond the initial application is not well characterized (Straw et al. 2022). Unlike pesticide active ingredients, many adjuvant principal functioning agents and formulation inerts have exemptions to food residue tolerances in the United States, so there is little incentive to improve environmental degradation for the exempted compounds. All 5 of the main chemical classes and all main functions of adjuvant principal functioning agents include compounds with tolerance exemptions (USEPA 2015, 40 CFR Part 180 2023). Trisiloxane organo-silicone adjuvants are not environmentally persistent, as they are not commonly found in air, rapidly degrade in water, and do not readily travel from plant roots to leaves and flowers, so the only known exposure route to honey bees is through direct spraying of foraging bees or through contact with pollen that has been sprayed directly (Slade 2020). Direct spray exposure concentration can vary between 0.0625 and 0.625 percent by volume, depending on the labeled application rate of the adjuvant of interest (Shannon et al. 2023). In a study of trisiloxane organo-silicone adjuvant residues in hives, these adjuvants were found in every wax sample and 60% of pollen samples, though none were found in honey (Chen and Mullin 2013, Mullin 2015). Residue analysis of trisiloxane adjuvants following applications on almonds (Collins and Jackson 2022) detected residue levels as high as 76.2 ppm in almond pollen, and as high as 2.12 ppm in almond nectar. The concentrations of trisiloxane adjuvants in hive products were as high as 0.177 ppm in stored nectar, 40.5 ppm in bee bread, and 12.1 ppm in wax. The maximum average concentration in larvae was 12.1 ppm and in adult bees was 8.32 ppm. Using the BeeREX model (USEPA 2014), the authors determined that the adjuvant exposure in this study posed a low risk for causing honey bee mortality (Collins and Jackson 2022). However, adjuvants have the potential to be mobile and may appear in surface or groundwater sources used by bees and other beneficial organisms (Tu and Randall 2003). Some ethoxylate surfactants can have a half-life of up to 6 mo in water where they can cause a reduction in surface tension leading to honey bee drowning at concentrations as low as 25 ppm (Moffett and Morton 1973, 1975, Straw et al. 2022). Although concentrations expected to cause an effect are unlikely in large water bodies where pesticide runoff would be diluted, it is plausible that surfactants in small ponds, puddles, or irrigation ditches could cause honey bee mortality (Moffett and Morton 1973). Foraging bees that are directly sprayed with a pesticide application or bees collecting water from affected water bodies are most likely to be exposed. Forager bees are known to be generally more sensitive to pesticides than in-hive bees, possibly due to their lower weight or protein deficiency due to decreased pollen intake (Wahl and Ulm 1983, Vance et al. 2009, Tosi and Nieh 2019). Therefore, foragers are likely to be both most susceptible and most likely to receive direct exposure to adjuvants and adjuvant-pesticide tank mixtures.

## Effects of Adjuvants on Honey Bees

Generally, adjuvant products do not claim any pesticidal activity, though Tergitol, a commercially available alcohol ethoxylate, is registered for use as a mosquito larvicide through reduced water surface tension to cause death of larvae through drowning (Ware and Whitacre 2004). However, some adjuvants that are not registered as pesticides have been recognized for their utility in controlling insects and other arthropods in the absence of any insecticide active ingredient. Nonionic and anionic adjuvants have demonstrated potential for control of aphids (Wolfenbarger 1964, Radwan et al. 1982, Cloyd 2005), spider mites (Searle et al. 1990), and cockroaches (Sims and Appel 2007).

Although bee toxicity testing is not required for adjuvants and other products defined by USEPA as inert ingredients (USEPA 2013c), concerns regarding bee toxicity have been raised (Mullin 2015). A study from New Zealand showed that adjuvant products can cause acute bee mortality in contact and oral laboratory bioassays at field-relevant concentrations (Goodwin and McBrydie 2000). Polyethoxylates have raised concern about their toxic effects on multiple bee species at or below field use rates, or up to 0.625% concentration by volume (Krogh et al. 2003, Artz and Pitts-Singer 2015, Mullin 2015, Mullin et al. 2015, Ricke et al. 2021, Walker et al. 2022, Shannon et al. 2023). Organo-silicone surfactants have also raised concerns about their toxic effects on bees with doses as low as 0.550 ppm in ingested sugar water or 18 µg per bee in topical applications. (Moffett and Morton 1973, Ciarlo et al. 2012, Chen and Mullin 2013, Mullin et al. 2015, Fine, Cox-Foster, et al. 2017, Chen et al. 2018, Durant et al. 2020, Slade 2020, Ricke et al. 2021, Walker et al. 2022, Wernecke et al. 2022, Shannon et al. 2023). Chronic exposure to Sylgard 309, a trisiloxane organo-silicone, caused mortality and delayed pupal eclosion (Fine, Cox-Foster, et al. 2017) and has also been shown to cause mortality in a beneficial wasp, the aphid parasitoid *Diaeretiella rapae* (Acheampong and Stark 2004). Pyrrolidone emulsifiers such as N-methyl-2-pyrrolidone can cause mortality in adult honey bees (Zhu et al. 2014, Mullin 2015, Mullin et al. 2015, Fine and Mullin 2017) and can be at least 20 times more toxic to larvae than adult honey bees in chronic feeding assays (Fine and Mullin 2017, Fine, Mullin, et al. 2017). Pyrrolidones are also toxic to *Daphnia magna* (Lan et al. 2004). Exposure to alkylpyrrolidones and their metabolites can also have toxic effects in humans (Sasaki et al. 1991, Wu et al. 2001, Schindler et al. 2012).

In addition to causing mortality, exposure to adjuvants can cause sublethal effects in honey bees. Oral exposure to trisiloxane organo-silicone and alcohol ethoxylate adjuvants can impair honey bee learning (Ciarlo et al. 2012, Mullin 2015, Chen et al. 2018). However, no effect of adjuvants was found on floral preference (Moffett and Morton 1975). Formulated fungicides, alcohol ethoxylate adjuvants, and their combinations disrupt the nest recognition abilities of *Osmia* and *Megachile* solitary bee species (Artz and Pitts-Singer 2015). Fungicide-adjuvant combinations used in almond orchards showed no effect on queen development or survival (Johnson and Percel 2013), though the combination of an insecticide, fungicide, and adjuvant resulted in reduced queen development and survival (Ricke et al. 2021). In plants, the surfactant activity of adjuvants reduces the surface tension of water and facilitates the uptake of bacterial-sized mineral particles through the stomata; the same mechanism may facilitate the movement of pathogens into bee tissues (Kaiser 2014, Mullin 2015). Generally, sublethal concentrations of toxic substances can induce “malaise” behavior in honey bees at sublethal doses, causing increased grooming, reduced locomotion, abdomen dragging, curling up, and reduced ability to right themselves when knocked on their sides or back (Hurst et al. 2014). There is evidence that bees can detect and avoid some adjuvants as bees show a preference for clean water sources over those sprayed with adjuvants, though they will still visit water sources contaminated with adjuvants if other sources of water are insufficient or unavailable (Moffett and Morton 1973, 1975).

While the mechanism of action for adjuvant toxicity to insects is not well-understood and there may be multiple mechanisms depending on the class of adjuvant chemistry, it is likely that many amphiphilic adjuvants, when encountered at high concentrations, can work through a mechanism similar to insecticidal soaps, as both have similar properties and contain hydrophobic and hydrophilic functional groups (Cowles et al. 2000, Cirelli et al. 2010, Castro et al. 2014). Insecticidal soaps, made up of a dissolved cation and fatty acid (Ralston et al. 1941, Puritch 1975, Parry and Rose 1983, Baldwin et al. 2008), decrease water surface tension so that water readily penetrates insect spiracles, reducing oxygen availability and causing asphyxiation through drowning (Ware and Whitacre 2004, Yu 2008). Insecticidal soap applied to cockroaches does not cause mortality through tarsal contact and the tracheal system of cockroaches treated with dyed soap solutions show evidence of soap solution penetration (Baldwin et al. 2008), supporting the hypothesis that spiracular penetration is driving toxicity (Fulton 1930, Dills and Menusan 1935, Richards and Weygandt 1945, Szumlas 2002, Abbasi et al. 2013). Honey bees exhibit spiracular drowning in 1% sodium oleate soap solution but not with water alone (McGovran 1929, Moffett and Morton 1973). Organo-silicone adjuvants have caused asphyxiation in insects and other arthropods by water infiltration into spiracles through reduced water surface tension (Dentener and Peetz 1992, Stevens 1993). It is thought that asphyxiation through spiracular downing is the most likely mechanism of adjuvant toxicity in spider mites (Cowles et al. 2000).

In addition to causing drowning, insecticidal soaps can disrupt the cuticle and break down cell membranes to cause rapid death in insects and mites (Ware and Whitacre 2004, Yu 2008). The fatty acids in insecticidal soaps can also exert hemolytic action, inhibit proteolytic enzymes, and cause immediate paralysis in aphids (Siegler and Popenoe 1925). Many surfactant adjuvants disrupt cell membranes by solubilizing the phospholipid bilayer (Borenfreund and Puerner 1985, Jones 1999, Weyermann et al. 2005) and are commonly used as positive controls in cytotoxicity studies of membrane disruption (Riss et al. 2019).

However, adjuvants may also work through other mechanisms to cause sublethal effects in bees. Oral administration of alcohol ethoxylate adjuvants to bumble bees (*Bombus terrestris audax*) caused midgut damage indicated by melanization, a reduction in appetite, weight loss, and increased mortality (Straw and Brown 2021). Octamethylcyclotetrasiloxane, a cyclic organo-silicone that does not have amphiphilic properties and is typically present in low (1% or less) concentrations in organo-silicone adjuvant formulations, has demonstrated reproductive toxicity and endocrine effects in mammals (Lassen et al. 2005, Quinn et al. 2007).

## Adjuvant Interactions with Pesticides and Other Bee Stressors can Increase Adverse Effects in Honey Bees

The expectation is that pesticide and adjuvant products combined in a tank mix will demonstrate additive toxicity in the absence of any chemical interactions. However, adjuvant-pesticide combinations may exhibit synergistic interactions, where the joint effect of 2 or more chemicals has a greater effect than predicted from concentration addition (Cedergreen 2014). Synergism between adjuvants and pesticides has been used in pest management of other arthropods, such as aphids (Acheampong and Stark 2004), spider mites (Dentener and Peetz 1992, Cowles et al. 2000), and thrips (Tang et al. 2022).

There is concern that these synergistic adjuvant-pesticide interactions in pesticide tank mixes may also increase mortality in honey bees and other beneficial organisms. A 2% concentration of the formulated adjuvant Dyne-Amic, which contains modified seed oils, organo-silicones, and alcohol ethoxylates, combined with the formulated insecticide Altacor causes increased toxicity to honey bees when tank mixed with certain fungicide products (Walker et al. 2022). A range of adjuvant products containing different chemical classes have demonstrated increased toxicity to honey bees when combined with individual pesticides (Shannon et al. 2023). Formulated organo-silicone adjuvants in tank mix combinations with formulated insecticide, fungicide, and herbicide products have also demonstrated synergistic toxicity to honey bees (Mullin et al. 2015, Wernecke et al. 2022). Adjuvants also demonstrate increased toxicity in mammalian cell lines when combined with the herbicide glyphosate (Coalova et al. 2014).

The mechanisms behind the synergistic interactions between adjuvants and pesticides are not well-understood. Combined mixtures of some formulated insecticides and fungicides, without additional adjuvants, have demonstrated synergistic toxicity to honey bees (Thompson and Wilkins 2003, Biddinger et al. 2013, Walker et al. 2022). Interactions between 2 formulated pesticides or between an adjuvant and a formulated pesticide could be caused by interactions between the active or the inert ingredients in the pesticide formulation. Synergism may be the result of increased spiracular or cuticular penetration due to the surfactant activity of the adjuvant, as observed in studies with adjuvants containing oil-based, nonionic, or organo-silicone components (Hurst 1940, Olson and O’Brien 1963, Lewis 1980, Dentener and Peetz 1992, Fanning et al. 2018). Adjuvants may increase permeability through insect cuticular waxes through the same mechanisms that allow increased ion permeability through the leaf cuticle (He and Rezai 2020). Other potential synergistic interactions could occur through interference with cytochrome P450-mediated detoxification, as some adjuvants, including N-methyl-2-pyrrolidine, may be metabolized by P450s (Fine and Mullin 2017). In cockroaches, an alcohol ethoxylate has been shown to synergize with piperonyl butoxide, a model P450 inhibitor, which indicates that tolerance of other adjuvants may be mediated by a P450 mechanism (Sims and Appel 2007). Honey bees exposed to a combination of an organo-silicone surfactant with the insecticide Altacor and the P450-inhibiting fungicide Tilt demonstrated a decrease in food consumption and increased transcription of cytochrome P450s (Thompson 2019). More work is needed to determine if other chemical classes of adjuvant principal functioning agents interact with P450s. Nonionic surfactants with amphiphilic properties can readily bind to proteins, peptides, and amino acids, and can modify membrane structure and permeability, which makes cytochrome P450 detoxification a potentially susceptible target site (Cserháti 1995).

There is also the potential for adjuvants to synergize with biotic threats to bee health, as adjuvants can increase the pathogenicity of bee viruses and mortality in larvae (Fine, Cox-Foster, et al. 2017, Chen et al. 2018). Adjuvants also can cause reduced food consumption and metabolic activity (Thompson 2019, Straw and Brown 2021). These effects of adjuvants have the potential to interact with other stressors, as insecticides can synergize with poor nutrition (Tosi et al. 2017) or other diseases (Alaux et al. 2010).

Antagonistic effects, where the joint effect of 2 or more chemicals is less than predicted by additive toxicity, is also possible in adjuvant-pesticide combinations (Cedergreen 2014). Adjuvants have demonstrated antagonism in tank mixtures with pesticides, both towards honey bees and towards the target pest. Formulated adjuvants from multiple chemical classes are less toxic to honey bees when sprayed in tank mix combinations with some formulated pesticides compared to the adjuvant alone (Shannon et al. 2023). Although the combination of the adjuvant product Dyne-Amic and the formulated insecticide Altacor demonstrated increased toxicity in combination with fungicides, the same study showed an antagonistic effect when Dyne-Amic was combined with a fungicide in the absence of insecticide (Walker et al. 2022). A formulation of the insecticide diflubenzuron demonstrated antagonism to the cabbage aphid *Brevicoryne brassicae* (L.) when combined with nonionic and anionic adjuvants (Radwan et al. 1982). Salts or other ions in spray carrier water have shown antagonism with herbicides (Nalewaja and Matysiak 1991, Nalewaja et al. 1991, Kirkwood 1993). Alcohol ethoxylates (Gaskin and Holloway 1992, Kirkwood 1993, van Toor et al. 1994) and organo-silicone adjuvants (Gaskin and Stevens 1993a, 1993b) have shown antagonism to glyphosate uptake in plants. Antagonism may be a result of solubility issues caused by a change in the pH of the spray solution, which can cause precipitates to form (Nalewaja and Matysiak 1991, Kirkwood 1993). It has been suggested that antagonism of glyphosate by organo-silicone adjuvants could be caused by the rapid spreading and drying of adjuvants, which would slow the rate of uptake into leaves over time (Gaskin and Stevens 1993a).

## Conclusion

Based on the current state of knowledge regarding adjuvant toxicity to honey bees, we recommend that pesticide applicators only add adjuvant products to pesticide tank mixes when recommended on the pesticide label. However, an adjuvant recommendation on a pesticide label does not imply that adjuvant-pesticide combinations were tested for bee safety but instead indicates that the pesticide manufacturer suggests use of an adjuvant to maximize efficacy of pest control. Adjuvants that are not recommended on pesticide labels can have unpredictable interactions with pesticides, such as synergistic interactions that may harm beneficial organisms or antagonistic interactions that reduce efficacy against the targeted pest. In addition to interactions affecting honey bees, the use of adjuvants when not recommended on the pesticide label can have negative effects on crop plants, such as reduced drought tolerance of crops (Räsch et al. 2018), altered physiochemistry of plant surfaces (Noga et al. 1986, Rogiers et al. 2005, Schreiber 2006, Damato et al. 2017) and induced phytotoxic effects (Knoche et al. 1992, Jursík et al. 2013). In addition to adjuvant-pesticide interactions, there is potential for synergistic interactions to occur between tank mixed pesticide products in the absence of an adjuvant (Thompson and Wilkins 2003, Biddinger et al. 2013, Johnson and Percel 2013, Walker et al. 2022), so caution should be taken when combining multiple products in a single application. If adjuvants are required, they should be applied at the minimum concentrations needed to achieve efficacy. Additionally, applicators should avoid the use of adjuvant products and pesticides containing inert ingredients known to have toxicity to honey bees. Bee exposure to adjuvants can be further reduced if pesticide applications are made when bees are not actively foraging, such as in the evening or at night, which is not a requirement for most adjuvant or pesticide products that do not carry bee hazard labeling. Growers should also use integrated pest management (IPM) strategies to reduce the use of pesticides in agricultural operations where honey bees and other pollinators may be present. Furthermore, our recommendation is against the use of adjuvant products in tank mixtures in IPM strategies due to the potential toxicity of these products against beneficial insects providing biocontrol of pests (Acheampong and Stark 2004).

Researchers should always clearly state whether the formulation or the active ingredient alone was used when reporting toxicities for pesticides and adjuvants. The use of an active ingredient name when testing a formulation containing inert ingredients results in confusion in the scientific literature that can lead to misunderstanding and inaccurate risk assessments for commercial pesticides and adjuvants (Mesnage and Antoniou 2018). More work is needed to characterize the mechanisms by which different inert ingredients affect bees, interactions between inerts with pesticide active ingredients, and interactions between inert ingredient toxicity with other bee stressors. Field trials may provide further insight as lethal effects of pesticides in field trials have been observed at lower concentrations than in lab testing (Fisher et al. 2021). Additionally, we recommend that researchers put more emphasis on exploring the adjuvants that possess low risk to honey bees, as all adjuvants are not equal in their bee toxicity (Shannon et al. 2023). Adjuvants have the potential to decrease the application rate of pesticide active ingredients by increasing their efficacy, which could improve environmental safety. While use of highly toxic adjuvants could cause the attempt at reduced environmental risk to backfire, adjuvants that can increase efficacy against the target pest while decreasing the impact on nontarget organisms have the potential to be beneficial from both a pest control and environmental perspective. Development of adjuvants that are mutually beneficial for pest control and environmental safety should be pursued to replace adjuvants with higher environmental risk.

The current state of research suggests that adjuvant and pesticide manufacturers should avoid the more toxic principal functioning agents in adjuvants and inert ingredients in pesticide formulations that will be applied on insect-pollinated crops, including some polyethoxylate and organo-silicone surfactants. If alcohol ethoxylates are used, it is recommended that compounds with fewer molar equivalents of ethoxylate are used, as contact toxicity in other insect species was inversely related to levels of ethoxylation (Saucedo-Pompa et al. 2022, Sims and Appel 2007). With the great diversity of compounds used as principal functioning agents in adjuvants, a concerted effort among honey bee researchers may be necessary to provide toxicological profiling of these inert ingredients. We recommend that both industry and regulatory agencies work to improve the disclosure of the identity and concentration of formulation components for pesticide and adjuvant products (Mullin et al. 2016). Additionally, more bee toxicity testing for pesticide formulation inert ingredients and adjuvants (Poquet et al. 2014, Suryanarayanan 2015) should be performed to better assess the risk of these compounds to pollinators and other nontarget species in addition to QSAR modeling (Mullin et al. 2016, Manibusan et al. 2023).

As the literature summarized in this review demonstrates (Table 1), the term “inert ingredients” misrepresents the chemical and biological activity of these compounds. Instead, we propose that these compounds should be classified as “co-formulant” or “supporting” ingredients to better reflect the chemical or biological effects of these compounds when they are included in pesticide formulations or adjuvant products (Tu and Randall 2003). It is important to dispel the misperceptions that are implied with the term “inert” in order for pesticide applicators to more accurately perceive the risks these compounds pose (Bostrom et al. 1992, Maharik and Fischhoff 1992, Atman et al. 1994, Morgan et al. 2002, Shannon et al. 2023).

**Table 1.** Summary of published routes of exposure and toxic effects of adjuvants to honey bees. There is currently no published data on the toxicity of individual components from the adjuvant chemical classes of fatty acid ethoxylates, modified seed oils, crop oil concentrates, or hydrocolloid polymers. “Commercial product tested” indicates the specific adjuvant product that was used in the study, but other commercial products may contain the same compound(s) of interest. Mention of company or trade names is for description only and does not imply endorsement by the USDA

Principle functioning agent group	Compound subclass	Commercial product tested	Routes of Exposure	Effect to Honey bees and other insects	Concentrations of toxicity	Source
Adjuvant (not specified)	N/A	N/A	Potential to be mobile and may appear in surface or groundwater sources	Not tested	Not listed	(Tu and Randall 2003)
Nonionic surfactant	Alcohol ethoxylate	C16–18 alcohols, ethoxylated (CAS-No. 68439-49-6)	Laboratory oral exposure	Gut damage in bumble bees indicated by melanization of the midgut, reduction in appetite, weight loss, and 30% mortality	0.16 ug per bee	(Straw and Brown 2021)
		Multi-Film X-77	Affected surface water sources	Drowning	25 ppm	(Moffett and Morton 1973)
		Multi-Film X-77; Dow Corning Fluid 471A	Half-life of 6 mo in water sources	Repellency to affected water sources	500 ppm	(Moffett and Morton 1975)
Nonionic surfactant	Alcohol ethoxylate	Tergitol	Application onto mosquito larval sources	Drowning of mosquito larvae	Not specified	(Ware and Whitacre 2004)
		Tomadol 23-1	Laboratory contact exposure (microapplicator)	Mortality in cockroaches and synergism with piperonyl butoxide, a cytochrome P450 inhibitor	2 uL per insect of 1.828 M concentration	(Sims and Appel 2007)
	Nonylphenol ethoxylate	N-90	Residual plant exposure	Adjuvant alone and adjuvant-fungicide combinations disrupted nest recognition abilities of *Osmia* and *Megachilid* solitary bee species	0.4 kg/ha	(Artz and Pitts-Singer 2015)
	Alkylphenol ethoxylate	Citowett	Laboratory contact (Potter Spray Tower) exposure	Contact (spray) mortality	Lowest active concentration 0.04%	(Goodwin and McBrydie 2000)
Nonionic surfactant	Alkyl polyethoxy ethers, ethoxylated derivatives, and isopropanol	Boost	Laboratory contact (Potter Spray Tower) and oral exposure	Contact (spray) and oral mortality	Lowest active concentration 0.01%	(Goodwin and McBrydie 2000)
	Tallow amine ethoxylate	Ethokem	Laboratory contact (Potter Spray Tower) and oral exposure	Oral and contact (spray) mortality	Lowest active concentration 0.5%	(Goodwin and McBrydie 2000)
Organo-silicone surfactants	Trisiloxanes	Not listed	Only known exposure route to honey bees is through direct spraying of foraging bees or through contact with pollen that has been sprayed directly	N/A	Not listed	(Slade 2020)
Organo-silicone surfactants	Trisiloxanes	Dyne-Amic, Kinetic HV, Silwet L-77, Sylgard 309	Found in every beeswax sample and 60% of pollen samples, though none were found in honey	N/A	Up to 390 ng/g in wax and 39 ng/g in pollen; average 116 ng/g in wax and 18 ng/g in pollen	(Chen and Mullin 2013, Mullin 2015)
		Trisiloxane‐317, Trisiloxane-OH, Trisiloxane-acetoxy	Residue levels in almond pollen posed a low risk for causing honey bee mortality	N/A	Up to 76.2 mg/kg in pollen	(Collins and Jackson 2022)
		Sylgard 309	Laboratory oral exposure	Chronic mortality, delayed pupal eclosion, and synergism with viral pathogenicity	10 ppm	(Fine, Cox-Foster, et al. 2017)
Organo-silicone surfactants	Trisiloxanes	Xiameter OFX-0309	Laboratory oral exposure	Upregulation of cytochrome P450 gene in response to organo-silicone in combination with Tilt fungicide and Altacor insecticide	1 ppm organo-silicone, 3 ppm Altacor insecticide, and 150 ppb Tilt fungicide	(Thompson 2019)
Organo-silicone surfactants	Organomodified polydimethyl siloxane	Pulse	Laboratory contact (Potter Spray Tower) and oral exposure	Contact (spray) and oral mortality	Lowest active concentration 0.02%	(Goodwin and McBrydie 2000)
		Syl-Tac; Sylgard 309; Silwet L-77	Laboratory oral exposure	Impaired honey bee learning through proboscis extension reflex	1%	(Ciarlo et al. 2012)
Pyrrolidone emulsifiers	Polyoxyethylene polymethylsiloxane	Dow Corning Fluid 471A	Half-life of 6 mo in water sources	Repellency to affected water sources	500 ppm	(Moffett and Morton 1975)
	N-methyl-2-pyrollidine	N/A; considered an inert ingredient in pesticide formulations	Laboratory oral exposure	50% mortality to honey bee larvae after feeding for 4 days	100 mg/liter	(Zhu et al. 2014)
				50% mortality to adult honey bees after feeding for one day	10,000 mg/liter	(Zhu et al. 2014)
				More toxic to larvae than adult honey bees in chronic feeding assays due to lower cytochrome P450 enzyme activity	2,000 ppm adult and 100 ppm larvae	(Fine and Mullin 2017, Fine, Mullin, et al. 2017)
Formulation containing multiple principal functioning agent classes	Alkylphenol ethoxylate, alcohol ethoxylate, and tall oil fatty acid	Activator 90	Laboratory contact (Potter Spray Tower) exposure	Topical spray application caused mortality at near field application rates	LC_50_ estimate = 14.8% concentration	(Shannon et al. 2023)
	Propionic acid, ammonium salt, alkylphenol ethoxylate phosphate ester, 2-hydroxy-1,2,3-propanetricarboxylic acid, ammonium sulfate, acrylic acid polymer sodium salt	Choice Weather Master	Laboratory contact (Potter Spray Tower) exposure	Topical spray application caused mortality at high concentrations greater than field use rates	LC_50_ estimate greater than 15% concentration	(Shannon et al. 2023)
	Alkanolamide surfactants, alkylaryl polyethoxyethanol sulfates, and 1,2-propanediol	Cohere	Laboratory contact (Potter Spray Tower) exposure	Topical spray application caused mortality at near field application rates, decreased toxicity when combined with individual insecticides or fungicides	LC_50_ estimate = 3.1% concentration	(Shannon et al. 2023)
Formulation containing multiple principal functioning agent classes	Methyl esters of C16–C18 fatty acids, polyalkylene oxide modified polydimethylsiloxane, Alkylphenol ethoxylate	Dyne-Amic	Laboratory oral exposure	Oral exposure can impair honey bee learning	1%	(Ciarlo et al. 2012)
			Laboratory oral exposure of nurse bees to pollen	Combinations with Altacor (chlorantraniliprole) insecticide and Tilt (propiconazole) fungicide caused 30% reduction in queen survival	0.8% adjuvant concentration	(Ricke et al. 2021)
			Laboratory contact (Potter Spray Tower) exposure	Dyne-Amic alone and combinations with Altacor (chlorantraniliprole) insecticide and Tilt (propiconazole) fungicide causes mortality at field application rates	2% adjuvant concentration	(Walker et al. 2022)
Formulation containing multiple principal functioning agent classes	Methyl esters of C16–C18 fatty acids, polyalkylene oxide modified polydimethylsiloxane, alkylphenol ethoxylate	Dyne-Amic	Laboratory contact (Potter Spray Tower) exposure	Topical spray application caused mortality at near field application rates, decreased toxicity when combined with individual insecticides or fungicides	LC_50_ = 8.4% concentration	(Shannon et al. 2023)
Formulation containing multiple principal functioning agent classes	Alkyl aryl polyoxylkane ethers and free fatty acids	Induce	Laboratory contact (Potter Spray Tower) exposure	Topical spray application caused mortality at near field application rates, increased toxicity when combined with individual insecticides or fungicides	LC_50_ = 4.1% concentration	(Shannon et al. 2023)
	Polyalkyleneoxide modified polydimethylsiloxane and nonionic surfactants	Kinetic	Laboratory contact (Potter Spray Tower) exposure	Topical spray application caused mortality at near field application rates, increased toxicity when combined with individual insecticides or fungicides	LC_50_ = 4.4% concentration	(Shannon et al. 2023)
Formulation containing multiple principal functioning agent classes	Phosphatidylcholine, methylacetic acid, and alkyl polyoxyethylene ether	LI-700	Laboratory contact (Potter Spray Tower) exposure	Topical spray application caused mortality at high concentrations greater than field use rates, pesticide-dependent increased or decreased toxicity when combined with individual insecticides or fungicides	LC_50_ = 22.7% concentration	(Shannon et al. 2023)
	Lecithin, methyl esters of fatty acids, and alcohol ethoxylate	Liberate	Laboratory contact (Potter Spray Tower) exposure	Topical spray application caused mortality at near field application rates	LC_50_ = 7.1% concentration	(Shannon et al. 2023)
	Alkylphenol ethoxylate, polyethylene glycol, tall oil fatty acids	Surf-90	Laboratory contact (Potter Spray Tower) exposure	Topical spray application caused mortality at near field application rates, decreased toxicity when combined with individual insecticides or fungicides	LC_50_ = 4.5% concentration	(Shannon et al. 2023)

## Data Availability

No original data was produced for this manuscript.
